# Management of unexpected placenta accreta spectrum cases in resource-poor settings

**DOI:** 10.1016/j.xagr.2023.100191

**Published:** 2023-04-01

**Authors:** Rozi Aditya Aryananda, Albaro José Nieto-Calvache, Johannes J. Duvekot, Aditiawarman Aditiawarman, Marcus J. Rijken

**Affiliations:** 1 Department of Obstetrics and Gynecology, Dr. Soetomo General Academic Teaching Hospital, Universitas Airlangga, Surabaya, Indonesia (Drs Aryananda and Aditiawarman); 2 Department of Obstetrics and Gynaecology, Erasmus University Medical Center, Rotterdam, The Netherlands (Drs Aryananda and Duvekot); 3 Clínica de Acretismo Placentario, Fundación Valle del Lili, Cali, Colombia (Dr Nieto-Calvache); 4 Department of Obstetrics and Gynaecology, Amsterdam University Medical Centers, Amsterdam, The Netherlands (Drs Nieto-Calvache and Rijken); 5 and Julius Global Health, University Medical Center Utrecht, Utrecht University, Utrecht, The Netherlands (Dr Rijken).

**Keywords:** maternal death, placenta accreta spectrum, resource-poor setting, surgery, unexpected

## Abstract

**BACKGROUND:**

On a global scale, cases of placenta accreta spectrum are often just identified during cesarean delivery because they are missed during antenatal care screening. Routine operating teams not trained in the management of placenta accreta spectrum are faced with difficult surgical situations and have to make decisions that may define the clinical outcomes. Although there are general recommendations for the intraoperative management of placenta accreta spectrum, no studies have described the clinical reality of unexpected placenta accreta spectrum cases in resource-poor settings.

**OBJECTIVE:**

This study aimed to describe the maternal outcomes of previously undiagnosed placenta accreta spectrum managed in resource-poor settings in Colombia and Indonesia.

**STUDY DESIGN:**

This was a retrospective case series of women with histologically confirmed placenta accreta spectrum treated in 2 placenta accreta spectrum centers after referral from remote resource-poor hospitals. Clinical outcomes were analyzed according to the initial type of management: (1) no cesarean delivery; (2) placenta left in situ after cesarean delivery; (3) partial removal of the placenta after cesarean delivery; and (4) post–cesarean hysterectomy. In addition, we evaluated the use of telemedicine by comparing the outcomes of women in hospitals that used the support of the placenta accreta spectrum center during the initial surgery.

**RESULTS:**

A total of 29 women who were initially managed in Colombia (n=2) and Indonesia (n=27) were included. The lowest volume of blood loss and the lowest frequency of complications were in women who underwent deferred cesarean delivery (n=5; 17.2%) and in those who had a delayed placental delivery (n=5; 20.7%). Five maternal deaths (14%) occurred in the group that did not receive telehelp, and 4 women died of irreversible shock because of uncontrolled bleeding.

**CONCLUSION:**

Previously undiagnosed placenta accreta spectrum in resource-poor hospitals was associated with a high risk of maternal mortality. Open–close abdominal surgery or leaving the placenta in situ seem to be the best choices for unexpected placenta accreta spectrum management in resource-poor settings. Telemedicine with a placenta accreta spectrum center may improve prognosis.

**AJOG Global Reports at a Glance**

**Why was this study conducted?**

This study was conducted to describe maternal outcomes of unexpected placenta accreta spectrum (PAS) intraoperatively in resource-poor settings.

**Key findings**

For the management of unexpected PAS in resource-poor hospitals, better outcomes were reported in cases where further abdominal surgery was abandoned and the woman referred, or the placenta was left in situ, compared with cases where attempts to remove the placenta or hysterectomy were undertaken. Intraoperative telehelp may provide intraoperative strategies to reduce complications during management of PAS in resource-poor settings.

**What does this add to what is known?**

This study confirms the high burden of mortality in women with PAS in resource-poor settings. Intraoperative management strategies are needed for cases with unexpected PAS, and this study described maternal outcomes regarding several surgical strategies, and the importance of intraoperative telehelp, which can be used to provide PAS management recommendations for resource-poor hospitals.

## Introduction

Placenta accreta spectrum (PAS) is one of the most frightening obstetrical emergencies, mostly leading to massive blood loss with major morbidity and even mortality.^1^ Expert opinion is that PAS should be managed in expertise centers.^2^ However, such centers may not exist or are far away for many clinicians working in resource-poor countries. In addition, PAS remains undiagnosed during antenatal care in up to half of the cases.^3^ In many cases, clinicians are confronted with an unexpected intraoperative diagnosis of PAS, which can lead to potentially life-threatening conditions when the surgical team and/or the hospital setting are not prepared for this emergency.

The complex situation of too many and, at the same time, too few cesarean deliveries (CDs) is especially valid in many low- and middle-income countries (LMIC): the number of CDs is rising,^4^ so it can be anticipated that the incidence of PAS will rise as well. In contrast to high-income countries, LMIC settings report insufficient availability of trained centers that provide expert management of PAS,^5^ although such centers are crucial in the prevention of maternal deaths related to this disease.^6^

Management guidelines are applicable to these PAS centers^2^^,^^7^ and to the unexpected intraoperative finding of PAS during a CD,^8^ but there are few publications that describe recommendations for the management of women diagnosed with unexpected PAS in LMIC settings. Furthermore, no studies have evaluated the clinical results of women with PAS initially managed at hospitals with poor resources.

We have set up PAS referral centers in Colombia and Indonesia, and a care regionalization program for women with PAS has been implemented since 2016. Interdisciplinary groups in charge of managing PAS (PAS teams) have established contact with hospitals in the region, offering advisory services on the diagnosis and treatment of PAS that meet the criteria applied in centers of excellence for PAS.^8^ A “telehelp” service has been established, with permanent 24/7 doctor–doctor interaction through phone calls and free or low-cost virtual platforms.^9^^,^^10^

This interaction also facilitates the transfer of women between hospitals and includes support during the initial management of emergent cases, as well as feedback to remote hospitals on the clinical outcomes of women transferred for surgery in the PAS centers.

This study aimed to investigate the maternal outcomes of unexpected PAS initially managed in a resource-poor hospital and referred to our PAS referral centers.

## Materials and Methods

This retrospective descriptive study included women with histologically confirmed PAS^11^ treated from 2016 to 2021 in 2 PAS centers in LMICs: the Dr. Soetomo General Academic Teaching Hospital, Universitas Airlangga, Surabaya, Indonesia (SGATH), and Clínica de Acretismo Placentario, Fundación Valle del Lili, Cali, Colombia (CAP).^5^^,^^12^ A resource-poor setting was defined as a hospital with a low level of maternal healthcare, in which it is possible to manage uncomplicated pregnancies, and detect, stabilize, and initiate management of unanticipated maternal–fetal problems and high-risk antepartum, intrapartum, or postpartum conditions, but not complex maternal–fetal conditions.^13^ We included all women with prenatally undiagnosed PAS who underwent CD in a resource-poor hospital and who, after initial management in that hospital, were transferred to the PAS center. The criteria for PAS centers included having multidisciplinary expertise and experience in the care of PAS. Such expertise may include maternal–fetal medicine; gynecologic surgery; gynecologic oncology; vascular, trauma, and urologic surgery; transfusion medicine; and intensivists, neonatologists, interventional radiologists, anesthesiologists, specialized nursing staff, and ancillary personnel.^8^ Seven cases were excluded because of incomplete documentation from the referring hospital (including the absence of histologic confirmation of PAS in the case of hysterectomy).^7^^,^^11^

The intraoperative suspicion of PAS in the resource-poor hospital was based on surgical reports that had to mention at least 1 of the following items: (1) bluish appearance with neovascularization on the uterine surface, (2) placental tissue invading the uterine wall with neovascularization, and/or (3) difficult partial delivery of the placenta. The intraoperative confirmation in the PAS center was based on the guidelines of the International Society for Placenta Accreta Spectrum^14^ and the International Federation of Gynecology and Obstetrics (FIGO).^15^ All tissue samples obtained for histologic analysis and pathology reports from resource-poor hospitals or PAS centers were reviewed.

For the analysis, women were divided according to the type of management in the initial hospital: (1) no CD and referral (laparotomy with subsequent abdominal wall closure and referral to the PAS center); (2) placenta left in situ after birth of the child and referral; (3) partial removal of the placenta after birth of the child and referral; and (4) referral after cesarean hysterectomy.

In addition, we evaluated the use of telehelp by comparing the outcomes of women in hospitals that used the support of the PAS center through telemedicine during the initial surgery.

### Definitions

The level of maternal care in the referral hospital was defined as basic (hospitals that only have an obstetrician, pediatrician, or general surgeon 24 hours a day, without an intensive care unit or other support specialties) or medium (hospitals that have an intensive care unit, but are not the regional referral center for serious obstetrical conditions).^13^

Previous blood loss was estimated by combining the measured blood loss in the suction machine with visual assessment by the previous surgeon and anesthesiologist in the initial hospital. Blood loss in the referral hospital was measured by collecting blood suction and gauze weights during surgery.

Intraoperative telehelp cases were defined as cases where the surgeon was seeking a second opinion from the PAS center to make clinical decisions during surgery in a resource-poor hospital.

Telehelp varied from confirmation of the clinical suspicion of intraoperative PAS to advice on surgical technique. The use of telehelp included phone calls, sending intraoperative photos, and video calls to provide intraoperative suggestions or surgical strategies to avoid unnecessary bleeding or organ damage. The advice given included instructions on open–close procedures, leaving the placenta in situ, avoiding dangerous maneuvers such as placental traction, performing bladder dissection, and placing multiple stitches in the colpouterine area to reduce the intraoperative bleeding. We dichotomized the use of telehelp and did not qualify it further.

#### Management of referred cases in the placenta accreta spectrum center

Women who arrived at the PAS center in a stable condition underwent abdominal ultrasonography to analyze topography and placental invasion. PAS management was dependent on the severity and location of placental invasion.^5^^,^^12^

In life-threatening situations, such as heavy vaginal bleeding and/or hemodynamic instability, emergency surgery was immediately performed with aortic clamping to reduce blood loss and simultaneous resuscitation, followed by intraoperative staging and surgical management, depending on the topography of the invasion and secondary bleeding control.^16^

### Study variables

Clinical and epidemiologic information was obtained from medical and laboratory records and from the PAS database of both PAS centers. The results of the histologic studies of the hysterectomy cases performed in hospitals with poor resources were collected.

### Statistical analysis

Continuous variables were expressed as central tendency measurements (mean and median) and dispersion measurements (standard deviation or interquartile range) based on normal distribution criteria. Categorical variables were expressed as absolute and relative frequencies. The findings were reported according to the STROBE (Strengthening the Reporting of Observational Studies in Epidemiology) guidelines (Supplemental File).

### Ethics

The study was registered under numbers 1711-2019 and 016-2022, respectively, and approved by the institutional review boards of the SGATH and CAP, and the boards of directors of the resource-poor hospitals. Informed consent was not required because the rules defined in the Medical Research Involving Human Subjects Act did not apply to this study. Participants were not subject to procedures and were not required to follow rules of behavior. This study complied with the principles of the Declaration of Helsinki.

## Results

Thirty-six cases met the inclusion criteria, and 7 were excluded from the data analysis because of incomplete data, resulting in 29 cases for the analyses.

From the 29 cases of unexpected PAS (27 from Indonesia and 2 from Colombia), 4 outcome groups were established on the basis of the initial management at the resource-poor hospital:

1. In 5 women, the laparotomy wound was closed after the intraoperative/clinical diagnosis of PAS was made (open–close laparotomy), and the women were referred to the PAS center.
2. Six women underwent CD (fundal hysterotomy, cord ligation, and closure of the uterus without touching the placenta and leaving the placenta in situ) and were referred to the PAS center.
3. In 11 women, the surgeons attempted to remove the placenta after the birth of the child through lower uterine segment hysterotomy, cutting through the uterine wall and placental tissue. The surgeons attempted to control the bleeding as much as possible, closed the abdominal wall, and referred the women to the PAS center.
4. In 7 cases, the surgeons attempted to remove the placenta after birth of the infant, after which a hysterectomy was performed, the abdominal wall was closed, and the patient was referred to the PAS center.

Demographic data and maternal outcomes, according to the type of initial management, are presented in Table 1.

**Table 1:** Clinical characteristics of women with unexpected intraoperative placenta accreta spectrum diagnosis according to initial hospital management

Characteristics	Open–close abdominal surgery	Left the placenta in situ	Partial removal of the placenta	Hysterectomy
N (%)	N=5 (17.2%)^a^	N=6 (20.7%)^a^	N=11 (37.9%)^a^	N=7 (24.1%)^a^
Maternal age (y)^b^	34 (27–42)	31 (29–41)	30 (26–42)	35 (28–40)
Previous cesarean delivery^b^	1 (1–2)	2 (1–2)	1 (1–2)	1 (1–3)
Previous curettage^b^	1 (0–2)	0 (0–1)	0 (0–1)	1 (0–2)
History of antenatal ultrasound, n (%)	5 (100)	5 (83.3)	9 (81.8)	7 (100)
Emergency surgery in the basic hospital, n (%)	2 (40)	1 (16.7)	6 (54.5)	4 (57.1)
Level of maternal care in the previous hospital, n (%)				
Basic level^c^	3 (60)	4 (66.7)	8 (72.7)	4 (57.1)
Medium level^d^	2 (40)	2 (33.3)	3 (27.3)	3 (42.9)
GA at surgery (wk)^b^	37 (31–38)	38 (37–38)	36 (26–38)	36 (24–39)
Telehelp, n (%)	3 (60)	3 (50)	0	1 (14.3)
Fetal death, n (%)	0	0	1 (9.1)	1 (14.3)
Duration of the transport (h)^b^	2 (1–5)	4 (1–48)	5 (1–9)	2 (1–4)
Blood loss before referral (mL)^b^	50 (50–100)	500 (200–700)	3000 (800–5000)	4500 (2500–7000)
Blood loss in PAS center (mL)^b^	2000 (1250–3550)	1850 (400–13,000)	3000 (200–6550)	300 (200–6700)
Total number of PRBC (unit)^b^	3 (1–4)	3 (1–8)	6 (3–8)	5 (0–8)
Abdominal package in initial surgery, n (%)	0	1 (16.7)	3 (27.3)	6 (85.7)
Emergency surgery in the PAS center, n (%)	1 (20)	2 (33.3)	10 (90.9)	6 (85.7)
Clinical PAS by FIGO classification				
1	0	0	2	3
2	1	2	8	3
3A	3	2	1	0
3B	0	2	0	1
3C	1	0	0	0
Histopathology, n (%)				
Accreta	0	0	2 (18.2)	3 (42.9)
Increta	2 (40)	2 (33.3)	8 (72.7)	3 (42.9)
Percreta	3 (60)	4 (66.7)	1 (9.1)	1 (14.3)
Management in the PAS center, n (%)				
• One-step conservative reconstructive surgery	2 (40)	1 (16.7)	0	0
• Secondary procedures to achieve hemostasis^e^	0	0	1 (9.1)	7 (100)
• Hysterectomy	3 (60)	5 (83.3)	10 (90.9)	0
Complication				
Bladder injury, n (%)	0	1 (16.7)	5 (45.5)	1 (14.3)
Intraabdominal abscess, n (%)	0	1 (16.7)	1 (9.1)	1 (14.3)
Need for abdominal package in PAS center, n (%)	0	1 (16.7)	3 (27.3)	3 (42.9)
ICU admission >24 h, n (%)	3 (60%)	6 (100)	10 (90.9)	4 (57.1)
Maternal death, n (%)	0	1 (16.7)	3 (27.3)	1 (14.3)

*FIGO*, International Federation of Gynecology and Obstetrics; *GA*, gestational age; *ICU*, intensive care unit; *PAS*, placenta accreta spectrum; *PRBC*, packed red blood cells.

a The incidence per group

b Median (interquartile range)

c Hospitals that only have an obstetrician, pediatrician, and general surgeon 24 hours a day, without an ICU or other support specialties

d Hospitals that have an ICU, but are not the regional referral center for serious obstetrical conditions

e Any procedure performed at the PAS center to achieve hemostasis after initial failed management at the basic hospital. For example, trachelectomy after a subtotal hysterectomy with bleeding, hysterectomy after an initial surgery in which the placenta was removed without having controlled the bleeding, relaparotomy to control bleeding foci after hysterectomy with hemoperitoneum, etc.

During antenatal care, 90% (n=26/29) of the women underwent ultrasound examination. Despite these ultrasounds, PAS remained undiagnosed until CD in all women.

In 16 of 29 women (55%), a planned CD (no previous active vaginal bleeding) was scheduled. Two women delivered infants who died of intrauterine fetal death before surgery.

Women who had deferred CD had the best clinical results (lower volume of bleeding and frequency of complications) (Table 1). Women with the worst results were those who underwent hysterectomy at the referring hospital or those in whom the placenta was partially removed.

Intraoperative telehelp was performed in 7 cases: in 5 cases, an open–close procedure was recommended, and in 6 cases it was advised to leave the placenta in situ. This prevented massive bleeding and reduced the use of blood transfusions (Table 1). Table 2 describes the results of the telehelp task. The cases managed with telehelp services at the PAS center had fewer complications.

**Table 2:** Clinical characteristics of women with intraoperative diagnosis of placenta accreta spectrum according to telehelp use

Characteristics	Without telehelp N=22	With telehelp N=7
Maternal age (y)^a^	34 (26–42)	31 (27–42)
GA at surgery (wk)^a^	37 (24–38)	38 (31–39)
Emergency surgery in the basic hospital, n (%)	9 (40.9)	4 (57.1)
Fetal birthweight (g)	2550 (500–3300)	2900 (1685–3400)
Fetal death, n (%)	2 (9.1)	0 (0)
Estimated blood loss in basic hospital (mL)^a^	3000 (70–7000)	200 (50–2500)
Blood loss in PAS center (mL)^a^	2000 (200–13,000)	1250 (300–5000)
Total number of PRBC (unit)^a^	5 (1–8)	2 (0–4)
Abdominal package in previous surgery, n (%)	8 (36.4)	2 (28.6)
Emergency surgery in the PAS center, n (%)	16 (72.7)	3 (42.9)
Histopathology, n (%)		
Accreta	5 (22.7)	0 (0)
Increta	13 (59.1)	2 (28.6)
Percreta	4 (18.2)	5 (71.4)
Management in the PAS center, n (%)		
One-step conservative reconstructive surgery	2 (9.1)	1 (14.3)
Secondary procedures to achieve hemostasis^b^	7 (31.8)	1 (14.3)
Hysterectomy	13 (59.1)	5 (71.4)
Complication		
Bladder injury, n (%)	6 (27.3)	1 (14.3)
Intraabdominal abscess, n (%)	3 (13.6)	0 (0)
Need for abdominal package in PAS center, n (%)	7 (31.8)	0 (0)
ICU admission >24 h, n (%)	17 (77.3)	6 (85.7)
Maternal death, n (%)	5 (22.7)	0 (0)

*GA*, gestational age; *ICU*, intensive care unit; *PAS*, placenta accreta spectrum; *PRBC*, packed red blood cells.

a Median (interquartile range);

b Any procedure performed at the PAS center to achieve hemostasis after initial failed management at the basic hospital. For example, trachelectomy after a subtotal hysterectomy with bleeding, hysterectomy after an initial surgery in which the placenta was removed without having controlled the bleeding, relaparotomy to control bleeding foci after hysterectomy with hemoperitoneum, etc.

There were 5 maternal deaths (17%); telehelp was not used in any of these cases. Four women (80%) died because of irreversible shock after uncontrollable bleeding following the initial surgery in a resource-poor hospital. In 1 woman, the placenta was left in situ, and she was referred to the PAS center 3 days after hospitalization. She arrived at the PAS center 2 days after being referred because of the difficulty in accessing transportation where the resource-poor hospital and tertiary center are located on different islands. The patient died of severe sepsis 6 days after the initial surgery.

## Comment

### Principal findings

Our data showed that unexpected PAS could be life-threatening, especially in resource-poor settings; 14% of the women in this case series died of complications related to PAS. The clinical decisions of the surgeon in cases of unexpected PAS diagnosed during CD largely predict the clinical course of women. The worst decision was to attempt to remove the placenta in a setting without additional strategies to control bleeding (Table 1).

### Results in the context of what is known

Lack of knowledge about the dangers of PAS is a major factor contributing to maternal mortality.^6^ Any woman with a history of CD and a low-lying placenta should be seen by a senior healthcare provider or referred for obstetrical consultation in a PAS center. However, the high frequency of failure in detecting PAS during antenatal care^3^ makes it clear that every obstetrician should be prepared for an unexpected intraoperative finding such as PAS.

### Clinical implications

Obstetricians need to be instructed on what to do in unexpected PAS cases, and more importantly, they should know what interventions should be avoided.^10^ A bluish appearance (Figure, A) of the uterine wall with newly formed vessels (Figure, B) is an important intraoperative sign of advanced grading of PAS,^15^ which could be confirmed during telehelp. Intraoperative diagnosis of PAS could play a crucial role in improving maternal outcomes.^16^^,^^17^

**Figure:**
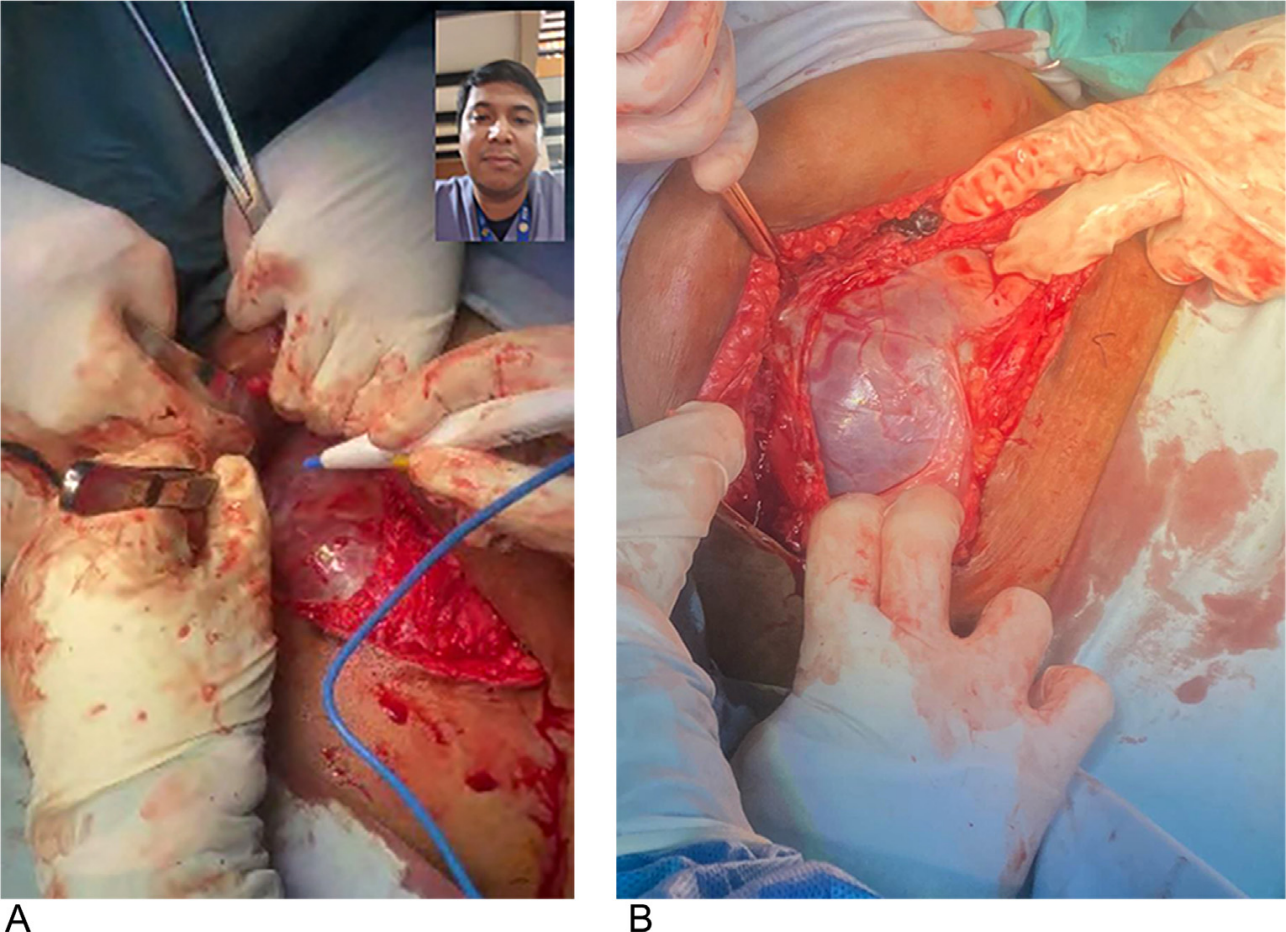
The importance of intraoperative telehelp for clinical diagnosis of PAS **A,** Intraoperative telehelp shows bluish appearance of the uterine wall. **B,** Pictures from a mobile phone may help for the intraoperative confirmation of placenta accreta spectrum, especially to detect newly-formed vessels.

Aryananda. Unexpected placenta accreta spectrum in resource-poor settings. Am J Obstet Gynecol Glob Rep 2023.

On the basis of the cases described in this series, not touching the placenta (opening and closing of the abdomen or placenta left in situ, and referral to a PAS center) is highly recommended to avoid massive bleeding during surgery at resource-poor hospitals.^18^ This observation coincides with expert opinion emphasizing the importance of deferring surgery until the recommended resources are available.^19^

In some situations, it is impossible to refrain from CD (because of fetal well-being or other indications for immediate delivery). In such cases, the fetus should be delivered, preferably using fundal hysterotomy, while avoiding and not touching the placenta and subsequently closing the uterus and abdomen, to refer the women to a hospital with experience in the management of PAS.

### Research implications

Damaging the placenta or placental removal without proper vascular control can lead to a critical condition of massive bleeding^7^^,^^20^; in this situation, aortic compression and seeking help may improve maternal outcomes.^21^

In some situations (for example, in women with severe vaginal bleeding), it may not be possible to postpone a hysterectomy in women with intraoperatively diagnosed PAS. In such situations, advice can be obtained through telemedicine.^9^ It is essential for each remote or basic hospital to have such a telehelp facility in place, and to ensure that it is embedded in the surgical management guidelines. With such a system, recommendations can be quickly and effectively applied.

Obstetricians caring for women with antenatal diagnosis of PAS should maximize the effort to preferably refer women to expert teams in a timely manner.^22^ Furthermore, all hospitals should prioritize the possibility of telecommunications, and the emergency setting of these cases.^23^ In this case series, intraoperative telehelp proved to be very useful for advice on surgical strategy. Sharing video calls and intraoperative photographs provided adequate support.^10^ Telehelp also helped to clarify which interventions should be avoided. None of the surgeons who decided to remove the placenta used telehelp, and only these women had the worst clinical results.

Table 2 suggests a relationship between the use of telehelp and lower volume of blood loss and number of blood transfusions. Telemedicine requires multiple formal processes before the interaction between the participating hospitals; however, doctor–doctor interaction has been recommended (maintaining the responsibility for the treatment with the doctor who is treating the PAS case) as a strategy to facilitate the application of this resource between hospitals in countries with different regulatory standards.^24^ Telehelp must be used not only to improve maternal outcome during surgery but also to improve the knowledge of the surgeon about surgical strategies for PAS that can be carried out, especially in the low-resource setting.

Although all cases included in this study were initially managed in basic resource-poor hospitals, the intraoperative finding of PAS can also occur in normal- or high-resource or referral hospitals that may not have a PAS team or where the PAS team is not available immediately. In line with the expert opinion,^2^ we recommend that if the condition of the mother and fetus allows it, surgery should be deferred (Table 3).

**Table 3:** Recommendations for undiagnosed placenta accreta spectrum in resource-poor settings

Recommendation to surgeons
1. Always look for a sign of PAS in the anterior wall of the uterus in pregnant women with previous cesarean delivery. 2. If there is a sign of PAS, avoid cutting through the placenta and extract the fetus through a fundal incision. 3. Before such an incision, call for help. Ask the hospital manager for a formal support mechanism in the obstetrical ward or during surgery. 4. Train easy and effective strategies to control pelvic bleeding (as internal manual aortic compression).

Recommendation to hospitals
1. Prepare a plan for massive obstetrical bleeding. 2. Define additional personnel to support the initial response team in the case of undiagnosed PAS during surgery. 3. Define a formal route for emergency transfer of pregnant women with intraoperative PAS finding. 4. Establish a formal telehealth process for severe obstetrical emergencies including PAS.

*PAS*, placenta accreta spectrum.

### Strengths and limitations

The limitation of this study is bias inherent to the nature of the study design. This was a retrospective study, and therefore some information was not available; for example, we were unable to identify why telehelp was not used in some cases, whether there were other contributing factors, or how many women were not referred in the study time period. However, this is a large case series of women with PAS as an unexpected intraoperative finding. Our observations draw attention to a relatively frequent situation among women with PAS (late diagnosis during laparotomy), and it is necessary to carry out multicenter studies at the regional or national level to evaluate the clinical results of women with an intraoperative diagnosis of PAS and the feasibility and utility of telemedicine in those situations.

### Conclusion

Unexpected PAS at CD in resource-poor hospitals increases the risk of maternal mortality. Open–close abdominal surgery and leaving the placenta in situ are the preferred choices for undiagnosed PAS management in such settings. Telehelp with a PAS center may improve prognosis by distinguishing PAS intraoperatively and advising on surgical strategies to reduce maternal mortality and morbidity.

## Appendix Supplementary materials

Image, application 1

## Footnotes

Supplementary material associated with this article can be found in the online version at doi:10.1016/j.xagr.2023.100191.
